# Targeted Genetic Screen in Amyotrophic Lateral Sclerosis Reveals Novel Genetic Variants with Synergistic Effect on Clinical Phenotype

**DOI:** 10.3389/fnmol.2017.00370

**Published:** 2017-11-09

**Authors:** Johnathan Cooper-Knock, Henry Robins, Isabell Niedermoser, Matthew Wyles, Paul R. Heath, Adrian Higginbottom, Theresa Walsh, Mbombe Kazoka, Project MinE ALS Sequencing Consortium, Ahmad Al Kheifat, Paul G. Ince, Guillaume M. Hautbergue, Christopher J. McDermott, Janine Kirby, Pamela J. Shaw

**Affiliations:** Maurice Wohl Clinical Neuroscience Institute, King's College London, Department of Basic and Clinical Neuroscience, London, United Kingdom; Maurice Wohl Clinical Neuroscience Institute, King's College London, Department of Basic and Clinical Neuroscience, London, United Kingdom; Suna and Inan Kirac Foundation, Neurodegeneration Research Laboratory, Bogazici University, Istanbul, Turkey; Faculty of Medicine and Health Sciences, Macquarie University, Sydney, NSW, Australia; Department of Neurology, Brain Center Rudolf Magnus, University Medical Center Utrecht, Utrecht, Netherlands; Academic Unit of Neurology, Trinity College Dublin, Trinity Biomedical Sciences Institute, Dublin, Republic of Ireland; Department of Neurology, Beaumont Hospital, Dublin, Republic of Ireland; Sheffield Institute for Translational Neuroscience (SITraN), University of Sheffield, Sheffield, United Kingdom; Biostatistics Department, Harvard School of Public Health, Boston, MA, United States; Department of Biostatistics, IoPPN, King's College London, London, United Kingdom; Department of Neurology, University of Massachusetts Medical School, Worcester, MA, United States; Department of Neurology, University of Massachusetts Medical School, Worcester, MA, United States; Population Genetics Laboratory, Smurfit Institute of Genetics, Trinity College Dublin, Dublin, Republic of Ireland; Maurice Wohl Clinical Neuroscience Institute, King's College London, Department of Basic and Clinical Neuroscience, London, United Kingdom; University of Exeter Medical School, Exeter University, St. Luke's Campus, Magdalen Street, Exeter EX1 2LU, United Kingdom; Department of Neurology, Brain Center Rudolf Magnus, University Medical Center Utrecht, Utrecht, Netherlands; KU Leuven - University of Leuven, Department of Neurosciences, Experimental Neurology and Leuven Research Institute for Neuroscience and Disease (LIND), B-3000 Leuven, Belgium; VIB, Vesalius Research Center, Laboratory of Neurobiology, Leuven, Belgium; University Hospitals Leuven, Department of Neurology, Leuven, Belgium; Hospital San Rafael, Madrid, Spain; Faculty of Medicine, University of Southampton, Southampton, United Kingdom; Department of Biostatistics, IoPPN, King's College London, London, United Kingdom; Biomedical Research Centre for Mental Health, IoPPN, King's College London, London, United Kingdom; Department of Neurology, Brain Center Rudolf Magnus, University Medical Center Utrecht, Utrecht, Netherlands; Maurice Wohl Clinical Neuroscience Institute, King's College London, Department of Basic and Clinical Neuroscience, London, United Kingdom; Maurice Wohl Clinical Neuroscience Institute, King's College London, Department of Basic and Clinical Neuroscience, London, United Kingdom; Maurice Wohl Clinical Neuroscience Institute, King's College London, Department of Basic and Clinical Neuroscience, London, United Kingdom; Department of Neurology, Brain Center Rudolf Magnus, University Medical Center Utrecht, Utrecht, Netherlands; KU Leuven - University of Leuven, Department of Neurosciences, Experimental Neurology and Leuven Research Institute for Neuroscience and Disease (LIND), B-3000 Leuven, Belgium, VIB, Vesalius Research Center, Laboratory of Neurobiology, Leuven, Belgium; University Hospitals Leuven, Department of Neurology, Leuven, Belgium; Department of Neurology, Brain Center Rudolf Magnus, University Medical Center Utrecht, Utrecht, Netherlands; Department of Neurology, Brain Center Rudolf Magnus, University Medical Center Utrecht, Utrecht, Netherlands; Department of Neurology, Brain Center Rudolf Magnus, University Medical Center Utrecht, Utrecht, Netherlands; Department of Neurology, Brain Center Rudolf Magnus, University Medical Center Utrecht, Utrecht, Netherlands; Department of Neurology, Brain Center Rudolf Magnus, University Medical Center Utrecht, Utrecht, Netherlands; Department of Neurology, Brain Center Rudolf Magnus, University Medical Center Utrecht, Utrecht, Netherlands; Department of Neurology, Brain Center Rudolf Magnus, University Medical Center Utrecht, Utrecht, Netherlands; SURFsara, Amsterdam, Netherlands; Emory University, Atlanta, United States; KU Leuven - University of Leuven, Department of Neurosciences, Experimental Neurology and Leuven Research Institute for Neuroscience and Disease (LIND), B-3000 Leuven, Belgium; VIB, Vesalius Research Center, Laboratory of Neurobiology, Leuven, Belgium; University Hospitals Leuven, Department of Neurology, Leuven, Belgium; Hadassah University Hospital, Jerusalem, Israel; Tel-Aviv Medical Center, Tel-Aviv, Israel; Faculty of Medicine and Health Sciences, Macquarie University, Sydney, NSW, Australia; Universidade de São Paulo, Brazil; Universidade de São Paulo, Brazil; Université de Limoges, France; Université François-Rabelais, Tours, France; IRCCS Instituto Auxologico Italiano, Milan, Italy; Universita degli Studi dei Torino, Turin, Italy; Instituto de Medicina Molecular, University of Lisbon, Lisbon, Portugal; Instituto de Medicina Molecular, University of Lisbon, Lisbon, Portugal; Hospital Carlos III, Madrid, Spain; Umeå University, Umeå, Sweden; Kantonspittal St. Gallen, St. Gallen, Switzerland; IRCCS Instituto Auxologico Italiano, Milan, Italy.; Sheffield Institute for Translational Neuroscience, University of Sheffield, Sheffield, United Kingdom

**Keywords:** amyotrophic lateral sclerosis, RNA binding proteins, oligogenic inheritance, *C9ORF72*, DNA sequencing

## Abstract

Amyotrophic lateral sclerosis (ALS) is underpinned by an oligogenic rare variant architecture. Identified genetic variants of ALS include RNA-binding proteins containing prion-like domains (PrLDs). We hypothesized that screening genes encoding additional similar proteins will yield novel genetic causes of ALS. The most common genetic variant of ALS patients is a G4C2-repeat expansion within *C9ORF72*. We have shown that G4C2-repeat RNA sequesters RNA-binding proteins. A logical consequence of this is that loss-of-function mutations in G4C2-binding partners might contribute to ALS pathogenesis independently of and/or synergistically with *C9ORF72* expansions. Targeted sequencing of genomic DNA encoding either RNA-binding proteins or known ALS genes (*n* = 274 genes) was performed in ALS patients to identify rare deleterious genetic variants and explore genotype-phenotype relationships. Genomic DNA was extracted from 103 ALS patients including 42 familial ALS patients and 61 young-onset (average age of onset 41 years) sporadic ALS patients; patients were chosen to maximize the probability of identifying genetic causes of ALS. Thirteen patients carried a G4C2-repeat expansion of *C9ORF72*. We identified 42 patients with rare deleterious variants; 6 patients carried more than one variant. Twelve mutations were discovered in known ALS genes which served as a validation of our strategy. Rare deleterious variants in RNA-binding proteins were significantly enriched in ALS patients compared to control frequencies (*p* = 5.31E-18). Nineteen patients featured at least one variant in a RNA-binding protein containing a PrLD. The number of variants per patient correlated with rate of disease progression (*t*-test, *p* = 0.033). We identified eighteen patients with a single variant in a G4C2-repeat binding protein. Patients with a G4C2-binding protein variant in combination with a *C9ORF72* expansion had a significantly faster disease course (*t*-test, *p* = 0.025). Our data are consistent with an oligogenic model of ALS. We provide evidence for a number of entirely novel genetic variants of ALS caused by mutations in RNA-binding proteins. Moreover we show that these mutations act synergistically with each other and with *C9ORF72* expansions to modify the clinical phenotype of ALS. A key finding is that this synergy is present only between functionally interacting variants. This work has significant implications for ALS therapy development.

## Introduction

Amyotrophic lateral sclerosis (ALS) is an age-related neurodegenerative disorder. The lifetime risk of ALS is ~1 in 400. The ALS phenotype is markedly variable but ~80% of patients die from respiratory failure within 2–5 years (Cooper-Knock et al., 2013). The majority of ALS is apparently sporadic, but 5–10% of patients show autosomal dominant inheritance. It is recognized that ALS is likely to be an oligogenic disorder even when it is apparently sporadic (van Blitterswijk et al., 2012). A mixed-model association analysis in 12,577 ALS cases and 23,475 controls was consistent with an oligogenic rare variant architecture (van Rheenen et al., 2016).

Identified ALS loci highlight a small number of pathways, most prominent of which is RNA metabolism. Pathogenic mutations have been discovered in multiple RNA-recognition motif (RRM) containing proteins including EWSR1, FUS, HNRNPA1, HNRNPA2B1, TAF15, and TDP-43 (Cooper-Knock et al., 2012). All of these proteins contain prion-like domains (PrLDs) (Harrison and Shorter, 2017). A PrLD consists of low complexity sequence with an “infectious” conformation that allows these proteins to undergo liquid-phase transition. Physiologically, such transitions allow the formation of membrane-less organelles such as stress granules, but pathologically they are thought to lead to irreversible protein aggregation. Often membrane-less organelles contain RNA; in addition to PrLD interaction it has been shown that RRM interaction with RNA is essential for integrity of so-called RNA granules (Molliex et al., 2015). The infectious aspect of PrLDs refers to the ability of aggregated protein to induce an aggregation–conformation in unaggregated protein, which is a proposed mechanism for ALS disease spread through the CNS (Ravits, 2014).

Thirty-one of the 213 identified RRM-containing proteins in the human proteome rank in the top 250 most prion-like (Alberti et al., 2009; Couthouis et al., 2011); this includes EWSR1, FUS, TAF15, and TDP-43 which are known to be mutated in ALS cases. We screened 147 additional genes encoding RRM-containing proteins with prion-like domains for mutations in ALS cases.

In the most common genetic variant of ALS, patients carry a G4C2-repeat expansion within intron 1 of *C9ORF72* (DeJesus-Hernandez et al., 2011; Renton et al., 2011). *C9ORF72*-ALS patients represent the full spectrum of sporadic ALS both clinically and pathologically (Cooper-Knock et al., 2012). The mechanism of pathogenesis in these cases is unknown. Three mechanisms have been proposed and to some extent demonstrated: (1) Haploinsufficiency related to disrupted expression of the *C9ORF72* protein. (2) Gain-of-function toxicity of G4C2-repeat RNA molecules transcribed from the mutated sequence. (3) Toxicity of dipeptide-repeat proteins translated from the repetitive RNA (Cooper-Knock et al., 2015b). It is hypothesized that G4C2-repeat RNA sequesters RNA-binding proteins away from their normal location causing a functional haploinsufficiency (Cooper-Knock et al., 2014). Notably the antisense transcript consisting of C4G2-repeat RNA binds a similar set of RNA-binding proteins (Cooper-Knock et al., 2015a). A logical consequence of this hypothesis is that loss-of-function mutations in G4C2-binding partners might contribute to ALS pathogenesis independently of and/or act synergistically with *C9ORF72* expansions. Evidence in myotonic dystrophy supports this hypothesis: mutations in muscleblind-like proteins modify the phenotype caused by sequestration of the same proteins by CUG-repeat RNA (e.g., Choi et al., 2016). Similarly mice lacking muscleblind-like 1 exhibit some of the features of myotonic dystrophy despite the absence of CUG-repeat RNA (Dixon et al., 2015).

We tested whether mutations in RNA-binding proteins, including both RRM-containing proteins with a PrLD and G4C2-binding partners, are a cause of ALS and/or whether they modify the clinical phenotype. Our patient cohort (Table 1, Supplementary Table 2) was comprised of either familial ALS cases caused by a *C9ORF72* expansion (*n* = 13) or FALS without a known genetic cause identified (*n* = 42) or young patients with sporadic ALS (*n* = 61) who are more likely to carry a pathogenic mutation than older patients with sporadic ALS (Cooper-Knock et al., 2013). Our filtering strategy aimed to identify rare deleterious variants rather than common low-risk variants. We also screened for variants in known ALS genes to augment the analysis and validate our strategy.

**Table 1:** Summary of targeted DNA sequencing screen.

**Group**	**Number of patients**	**Number with a newly identified variant**	**Number with >1 newly identified variant**	**Average age of onset (standard deviation) (years)**	**Male:Female Ratio**
Familial ALS	42	16	1	60 (8.6)	1.5:1
Young sporadic ALS	61	26	5	41 (15.8)	1.9:1
Total	103	42	6	49 (15.2)	1.8:1

We identified a number of apparently toxic variants in RNA-binding proteins in ALS patients at a significantly higher frequency than is observed in normal controls. Moreover we showed that these variants act synergistically with each other and with known ALS-causing mutations to determine the clinical severity of ALS. This has important implications for future ALS-therapy development.

## Materials and methods

### Design of the targeted genetic screen

The complete list of sequenced genes is provided in Supplementary Table 1. Genes were either known ALS genes or genes encoding RNA-binding proteins. The RNA-binding proteins were in two groups—RRM-containing proteins with a PrLD (Couthouis et al., 2011) or those identified binding partners of the G4C2-repeat expansion (Cooper-Knock et al., 2014).

### Selection of patients for screening

ALS patients were selected to increase the probability of discovering novel genetic variants—they either had a positive family history, or they were relatively young (<50 years old) at presentation or they carried an expansion of *C9ORF72*. Genomic DNA was extracted from 103 ALS patients from the North of England. The cohort included 34 familial ALS patients in whom a genetic cause had not been identified despite screening for ALS associated mutations in *SOD1, C9ORF72, TARDBP*, and *FUS*; 61 young-onset sporadic ALS patients; and thirteen *C9ORF72*-ALS patients (Table 1). A patient with an identified mutation in FUS was included as a positive control. G4C2-repeat expansions of *C9ORF72* expansions were identified by repeat-primed PCR as described previously (Cooper-Knock et al., 2012); all patients were screened for *C9ORF72* expansion prior to selection for the screen. The study was approved by the South Sheffield Research Ethics Committee and informed consent was obtained for all samples.

### DNA sequencing

Genomic DNA was enriched for selected RNA-binding proteins and known ALS genes using a custom designed Agilent SureSelect in solution kit. Sequencing was performed using an Illumina HiScan platform according to manufacturers instructions.

Rare deleterious mutations were defined by frequency within the Exome Aggregation Consortium data set of <1/10,000 control alleles (Lek et al., 2016), and a Phred-scaled Combined Annotation Dependent Depletion (CADD) score >10 (Kircher et al., 2014). Comparison of various pathogenicity prediction tools recently supported the sensitivity and specificity of CADD (Salgado et al., 2016). Given that we were focused on exonic changes with an effect on protein function, synonymous changes were excluded. We excluded any changes with a read depth <10 and validated by Sanger sequencing any changes with read depth 10–15 or a novel allele frequency less than one third the reference allele frequency (Supplementary Figure 1).

ExAC defines constrained genes based on an observed frequency of loss of function mutations which is much less than predicted by sequence specific mutation probabilities (Lek et al., 2016). A threshold for “constrained” is set as probability of a gene being loss of function intolerant (PLi) > 0.95.

## Results

Our aim was to identify genetic changes which may cause or contribute to ALS pathogenesis. Consistent with an oligogenic rare variant architecture of ALS (van Rheenen et al., 2016) we proposed that such changes are unlikely to be common in the background population, but may be present. We filtered sequencing data for rare deleterious variants defined as frequency within the ExAC data set of <1/10,000 control alleles (Lek et al., 2016), and a Phred-scaled CADD score >10 (change is within 10% most deleterious reference variants) (Kircher et al., 2014). All genetic changes with a low read depth were validated by Sanger sequencing (Supplementary Figure 1).

In 42 (of 103 screened) patients we identified a rare deleterious variant; six patients carried more than one variant. Thirteen *C9ORF72*-ALS patients were included in the screen; in eight we identified an additional rare deleterious variant (i.e., in addition to a G4C2-repeat expansion of *C9ORF72*) and in two patients we identified more than one additional variant. Average disease duration for patients in the screen was 66 months; average disease duration in patients with an identified variant was 61 months compared to 73 months in patients in whom no variant was identified, although this difference was not statistically significant (*t*-test, *p* = 0.14). In both patients with and without an identified variant average age of onset was 49 years.

### Identified mutations in known ALS genes

We identified 12 patients with mutations in nine known ALS genes (Table 2, Supplementary Table 2). This is expected based on reported frequencies of these mutations and served as a validation of our strategy. One patient with a previously identified FUS mutation was included as a positive control. Rare deleterious variants were newly identified in *ALS2, DCTN1* (two different variants), *ELP3, EWSR1, SETX* (two different variants)*, SOD1* (two different variants), *UNC13A, C9ORF72*, and *VCP*.

**Table 2:** Identified rare deleterious variants in known ALS genes.

**Gene**	**Mutation**	**Amino acid change**	**Mutated protein domain**	**Sporadic/Familial**	**CADD**
*C9ORF72*	A1239G	I413M	Alpha domain	Sporadic	13.9
*DCTN1*	G1326A/G1668A/G1617A/G1707A/G1728A	M442I/M556I/M539I/M569I/M576I	Dynein associated protein domain	Sporadic	17.2
*DCTN1*	G1193C/G1535C/G1484C/G1574C/G1595C	R398P/R512P/R495P/R525P/R532P	Dynein associated protein domain	Sporadic	24.6
*ELP3*	T654A/T795A/T735A/T969A/T1101A	Y218X/Y265X/Y245X/Y323X/Y337X	Affects all functional domains	Familial	37
*EWSR1*	G1366A/G1531A/G1534A/G1549A	G456R/G511R/G512R/G517R	Within R/G/P-rich domain	Familial	18.3
*SETX*	A6172C	K2058Q	Helicase domain	Familial	12.8
*SETX*	C1750G	L584V	Outside described domains	Familial	13.4
*UNC13A*	G3091A	G1031R	Calcium dependent secretion activator domain	Familial	11
*SOD1*	G217A	G72S	Cu/Zn binding domain	Sporadic	36
*SOD1*	T341C	I113T	Cu/Zn binding domain	Sporadic	19.5
*ALS2*	G1681A	V561I	Regulator of chromatin condensation domain	Sporadic	14.5
*VCP*	G278A	R93H	Aspartate decarboxylase-like domain	Sporadic	21.8

Several of the mutations we identified in ALS genes affect previously reported amino acids or protein domains. For example, both mutations in *DCTN1* were within the dynein associated protein domain, which is consistent with previously reported mutations (Münch et al., 2004); the mutation identified in *EWSR1* occurs in the same amino acid as previously reported (Couthouis et al., 2011); one of the *SETX* mutations we identified lies within a helicase domain which contains several previously reported mutations (Hirano et al., 2011); both *SOD1* mutations have been previously described in familial ALS (Orrell et al., 1999); and a mutation in the same amino acid of *VCP* has been previously identified in another ALS patient (Johnson et al., 2010).

Other variants we identified in known ALS genes are more novel. *ELP3* has been previously associated with ALS by GWAS (Simpson et al., 2009), but pathogenic variants have not been identified. The patient identified in this screen demonstrated a nonsense mutation in exon 10 which disrupts all described functional domains of the protein. Similarly variation in *UNC13A* has been identified as a risk factor for sporadic ALS (van Es et al., 2009) and as a modifier of the clinical phenotype, but pathogenic variants have not been identified. Our patient with a variant in *UNC13A* has a family history of ALS and no other identified mutation in an ALS gene (or any other gene in our screen). One sporadic ALS patient has a variant in *ALS2*; given that mutations in *ALS2* are usually autosomal recessive and associated with a slowly progressive juvenile onset form of the disease, then this variant is of unknown significance. However, no study has reported this exact change previously (Al-Chalabi et al., 2003; Luigetti et al., 2013). Similarly a rare deleterious variant was identified in *C9ORF72* in a patient who also carried a G4C2-repeat expansion; no pathogenic variants have been confirmed in *C9ORF72* except the G4C2-repeat expansion in intron 1 and therefore this variant is also of unknown significance.

### Identified rare deleterious variants in RRM-containing proteins with prion-like domains

**Figure 1:**
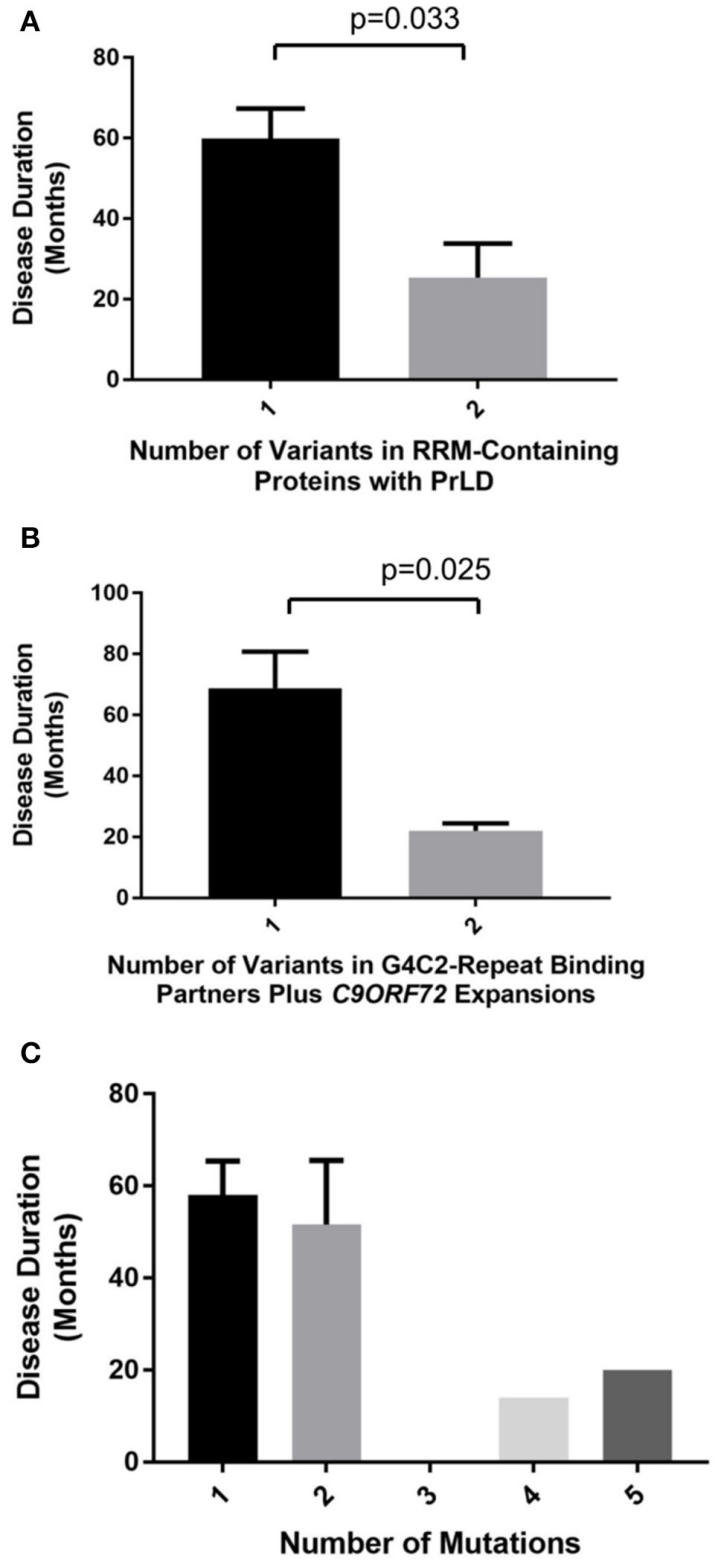
Number of identified rare deleterious variants in RNA-binding proteins is significantly correlated with ALS clinical phenotype only when variants are considered within functional subgroups. Plots show disease duration for ALS patients divided by number of identified rare deleterious variants. A significant relationship with ALS clinical phenotype is identified only when variants are considered within functional subgroups. ALS patients with two rare deleterious variants in RRM-containing proteins with PrLDs have a significantly faster disease course than patients with a single variant (*t*-test, *p* = 0.033) **(A)**. Rare deleterious variants in G4C2-binding partners lead to more rapid ALS disease progression when combined with a *C9ORF72* G4C2-repeat expansion (*t*-test, *p* = 0.025) **(B)**. In contrast when identified variants are considered together with mutations known ALS genes (including *C9ORF72* G4C2-repeat expansions) there is no significant correlation with disease phenotype **(C)**.

We identified 19 patients with a rare deleterious variant in a RNA-binding protein with a PrLD of whom three had more than one variant (Table 3, Supplementary Table 2). Fourteen of the patients had died, four patients were still alive and in these cases disease duration was censored to the present date. One patient with a variant in *MTHFSD* also had a mutation in *SOD1*. *SOD1* mutations are associated with a distinct clinical phenotype and pathology compared to characterized mutations in RNA-binding proteins (Cooper-Knock et al., 2013) and therefore this patient was excluded from further analysis. Of the 21 identified variants remaining, 16 (76%) occurred in either the RRM domain or a low complexity sequence (Table 3). The number of variants per patient correlated with rate of disease progression (Figure 1A, *t*-test, *p* = 0.033) but not age of onset. Including *C9ORF72* expansions in this analysis did not appear to be synergistic.

**Table 3:** Identified rare deleterious variants in RNA-binding proteins with prion-like domains.

**Gene**	**Variant**	**Amino acid change**	**Mutated RRM/Low complexity domain**	**Sporadic/Familial**	**Exac constrained (PLi > 0.95)**	**CADD phred score**	**Additional variant**
*NOL8*	T2597G/T2393G	L866R/L798R	E-rich domain	Sporadic	No	23.8	RBM4B
*RBM4B*	C701T	A234V	A-rich domain	Sporadic	No	15.2	NOL8
*EIF3B*	C943T	R315C	No	Familial	Yes	17.2	None
*RBM41*	G760A	A254T	No	Sporadic	No	11.3	None
*RBM12*	A622G	I208V	P-rich domain	Familial	No	12.4	RBM15
*RBM15*	G1787A	R596H	R-rich domain	Familial	Yes	20.6	RBM12
*HNRNPM*	G544A/G904A/G787A	G182S/G302S/G263S	No	Sporadic	Yes	22.7	None
*PPARGC1B*	C1037A/C962A/C1154A	P346H/P321H/P385H	No	Sporadic	No	12.1	None
*PPARGC1B*	A1648G/A1573G/A1765G	S550G/S525G/S589G	No	Sporadic	No	15.4	None
*PPARGC1B*	G1183A/G1108A/G1300A	E395K/E370K/E434K	E-rich domain	Sporadic	No	11.1	None
*MTHFSD*	G472C/G469C/G412C	A158P/A157P/A138P	No	Sporadic	No	22	None
*SPEN*	G1649A	R550H	RRM	Sporadic	Yes	34	None
*PABPC1L*	G808A	V270M	RRM	Sporadic	Yes	11.8	RBMXL3
*RBMXL3*	C362T	P121L	No	Sporadic	No	16.1	PABPC1L
*RAVER1*	T194G	L65R	RRM	Sporadic	Yes	28.3	None
*RBM12B*	A652T	M218L	RRM	Sporadic	No	17.9	None
*RBM15B*	G1385C	S462T	RRM	Sporadic	Yes	19.2	None
*RBM45*	G338A	R113Q	RRM	Sporadic	No	22.3	None
*RBMS2*	G354T	K118N	RRM	Familial	No	16.8	None
*RBMXL2*	G995T	R332L	R/E/P-rich domain	Sporadic	No	17.1	None
*TRNAU1AP*	G124T	G42W	RRM	Sporadic	No	29.6	None
*EWSR1*	G1366A/G1531A/G1534A/G1549A	G456R/G511R/G512R/G517R	Within R/G/P-rich domain	Familial	Yes	18.3	None

The Project MinE browser (http://databrowser.projectmine.com/) was utilized to search for additional evidence of similar variants in these proteins. The Project MinE Consortium has to date reported whole genome sequencing of 1169 ALS cases and 608 controls from the Netherlands. For *RBM4B, RBM45, RBMS2, RAVER1, PPARGC1B*, and *TRNAU1AP*, the project Project MinE data identified additional variant(s) within the same exon which were present either exclusively in ALS patients or were more frequent in ALS patients than controls. RBM12, *RBM12B, RBM15, RBM15B*, and *RBM45* are single exon genes, but Project MinE identified ALS cases with disease-associated variant(s) within <25 amino acids in each of these genes. This clustering of cases for each of these genes supports the functional significance of the rare variants we have discovered.

The ALS Variant Server, Worcester, MA (http://als.umassmed.edu/) reports whole exome sequencing from 1,022 familial ALS patients. Within this cohort we identified an additional example of an ALS patient carrying an A622G variant in *RBM12* and four ALS patients carrying p.S550G/p.S525G/p.S589G (single case) or p.E395K/p.E370K/p.E434K (3 cases) variants in *PPARGC1B*.

It is noteworthy that a small number of genes found to contain rare deleterious variants but classified as known ALS genes or G4C2-repeat binding partners are also RRM-containing proteins with a PrLD. This includes *EWSR1, HNRNPA3, HNRNPU*, and *HNRNPUL1*. Except for being previously identified as a known ALS gene, *EWSR1* is not distinct from the other RRM-containing proteins with a PrLD under consideration; therefore *EWSR1* is included in the analysis of synergy detailed above. In contrast *HNRNPA3, HNRNPU*, and *HNRNPUL1* were selected on the basis of an independent hypothesis: that loss of function in the proteins encoded by these genes might mimic sequestration by G4C2-repeat-RNA derived from a *C9ORF72* expansion. The majority of variants identified in RRM-containing proteins with a PrLD are located in either the RRM-domain or the PrLD but, consistent with an alternate mechanism, variants identified in *HNRNPA3, HNRNPU*, and *HNRNPUL1* are located distinct functional domains (Table 4). To avoid potentially confounding discrepancy between mechanisms of pathogenicity *HNRNPA3, HNRNPU*, and *HNRNPUL1* were not included in analysis of other variants identified within RRM-containing proteins with a PrLD.

**Table 4:** Identified rare deleterious variants in G4C2-repeat binding partners.

**Gene**	**Variant**	**Amino acid change**	**Sporadic/Familial**	**Exac constrained (PLi > 0.95)**	**CADD**	***C9ORF72* expansion**
*SLC1A3*	A509G/A647G	N170S/N216S	Sporadic	No	13.9	Yes
*SLC1A3*	C372G/C510G	F124L/F170L	Familial	No	10.1	No
*ATP5B*	T803C	V268A	Sporadic	Yes	25.5	No
*MYH9*	A3181T	S1061C	Sporadic	No	19.2	Yes
*EEF1G*	T1209A	D403E	Familial	Yes	14.5	Yes
*EEF1G*	C979T	R327C	Sporadic	Yes	18.3	No
*HNRNPUL1*	C161A	P54Q	Familial	Yes	11.8	No
*EPB41L3*	G1295A/G968A	R432H/R323H	Sporadic	No	29.2	No
*EZR*	C1714T	R572W	Sporadic	Yes	26.7	No
*GRSF1*	A364G	K122E	Familial	No	14.7	Yes
*HNRNPA3*	C35G	P12R	Familial	Yes	16.9	No
*HNRNPU*	C1202T/C1259T	S401L/S420L	Familial	Yes	35	Yes
*HSPA5*	G76A	D26N	Sporadic	No	18.6	No
*ILF3*	C1445T	S482L	Familial	Yes	12.4	No
*PA2G4*	A544G	I182V	Familial	Yes	11.2	No
*SRPK2*	G889C/G922C	A297P/A308P	Familial	Yes	20.4	No
*XRCC6*	T893C/T1043C/T920C	M298T/M348T/M307T	Sporadic	Yes	20	No
*XRCC6*	G1615A/G1765A/G1642A	G539R/G589R/G548R	Sporadic	Yes	11.2	No

### Identified rare deleterious variants in G4C2-repeat binding proteins

We identified 18 patients with a rare deleterious variant in a G4C2-repeat binding protein (Table 4, Supplementary Table 2). No patients had more than one variant in a G4C2-repeat binding protein. Five of the patients carried a G4C2-repeat expansion in *C9ORF72*. Fourteen of the patients had died, six patients were still alive and in these cases disease duration was censored to the present date. Patients with a G4C2-binding protein variant in combination with a *C9ORF72* expansion had a significantly faster disease course (Figure 1B, *t*-test, *p* = 0.025) but age of onset was not significantly different. For one patient with a variant in *ILF3*, no clinical information was available. In two specific examples the same gene is mutated in patents with and without a *C9ORF72* expansion—*SLC1A3* and *EEF1G*. In both cases there is a 50% reduction in disease duration (*SLC1A3*: 52 months to 27 months; *EEF1G*: 79 months censored to 22 months) in the patient carrying the *C9ORF72* expansion and the mutation.

Sequestration of RNA-binding proteins by G4C2-repeat RNA associated with *C9ORF72*-ALS would be expected to prevent those proteins performing their normal function. Consequently a mutation which exacerbates this toxicity would be expected to cause loss-of-function. Of the 18 G4C2-repeat binding proteins in which we identified a rare deleterious variant, 67% are encoded by genes which are defined by ExAC as extremely loss-of-function intolerant (ExAC refer to this property as “constrained”) (Lek et al., 2016). This is enriched compared to the total list of G4C2-repeat binding proteins screened (Supplementary Table 1) of which 42% are ExAC constrained (41 constrained from 98 total). This observation supports our proposed mechanism. In comparison, for RRM-containing proteins with a PrLD, the proportion of variants discovered in ExAC constrained genes is only 32%.

The Project MinE browser (http://databrowser.projectmine.com/) was utilized to search for additional evidence of similar variants in these proteins. For *SLC1A3, EEF1G, hnRNPU, hnRNPUL1, EZR*, and *GRSF1* Project MinE identified additional variant(s) within the same exon which were present either exclusively in ALS patients or were more frequent in ALS patients than controls. The ALS Variant Server, Worcester, MA (http://als.umassmed.edu/) reports whole exome sequencing from 1,022 familial ALS patients. Within this cohort we identified an additional example of an ALS patient carrying a p.D403E mutation in *EEF1G*, a p.P12R mutation in *HNRNPA3*, and a p.A297P/p.A308P mutation in *SRPK2*. This clustering of cases for each of these genes supports the functional significance of the rare variants we have discovered.

### Rare deleterious variants in RNA-binding proteins are enriched in ALS cases

To calculate whether the observed frequency of rare deleterious variants in RNA-binding proteins in our DNA sequencing screen is higher than expected we utilized ExAC frequencies and CADD scores for the identified changes. CADD scoring is expressed as the observed frequency of variants which are at least as pathogenic as the observed variant. For this analysis we assumed that observed frequency is independent of pathogenicity on the basis that ALS does not usually affect reproductive fitness. We observed 39 rare deleterious variants in 1,223,647 bases of DNA from 103 patients; this is a significant enrichment compared to observed control frequencies (*p* = 5.31E-18) suggesting that these variants are significantly enriched in ALS patients.

### Synergy between variants is function specific

No significant correlation was identified between total number of variants per patient and the clinical phenotype (Pearson correlation, correlation coefficient = −0.20, *p* = 0.21) (Figure 1C). This was unchanged whether or not *C9ORF72* expansions are considered. In contrast, when either RRM-containing proteins with PrLDs or G4C2 binding partners are considered in isolation, then there is a significant synergistic effect on clinical phenotype (Figures 1A,B). We conclude that a synergy is present only between variants in functionally interacting genes/proteins.

## Discussion

A new period of ALS genetics has begun in which we need to think of ALS as not a predominantly sporadic disease with a small proportion of monogenic familial cases, but rather as a pathogenesis shaped by synergy between oligogenic rare variants. It is likely that many ALS-associated genetic variants do not cause disease except in combination with other genetic and environmental factors. This is consistent with ALS as a multistep process as proposed by Al-Chalabi et al. (2014). With an oligogenic model in mind, we performed targeted genetic sequencing of RNA-binding proteins in ALS patients and identified rare deleterious variants at a significantly higher than control frequency. We aimed to identify novel pathogenic mutations and to discover evidence that these mutations act synergistically to produce the ALS phenotype. We achieved this and for the first time we have shown that synergy between mutations is specific to groups of functionally related genes/proteins.

We have shown that rare deleterious variants in RRM-containing proteins with a PrLD act synergistically to determine speed of ALS progression. Synergy is consistent with action in a common pathway. PrLD are thought to facilitate protein-protein interactions which are key to the formation of membrane-less cellular compartments (March et al., 2016). Important examples of membrane-less compartments are RNA-protein complexes such as P-bodies and stress granules. These RNA granules are dependent on protein-protein interaction via PrLDs in combination with protein-RNA interaction via RRMs (Harrison and Shorter, 2017). It is proposed that mutations in PrLDs or RRMs can affect this interaction and may increase the probability of transition to pathological aggregation. In support of this, a significant number of mutations already associated with ALS, which occur in RRM-containing proteins with PrLDs, cluster in or close to the PrLD or the RRM and make the protein more aggregation prone (Harrison and Shorter, 2017). A prediction of this model is that mutations in multiple proteins may act in synergy to produce aggregation. Consistent with this 76% of the variants we identified in RRM-containing proteins with PrLDs are within a low complexity sequence or a RRM.

We found that rare deleterious variants in G4C2-repeat-RNA binding partners act synergistically with *C9ORF72* expansions to shorten disease duration. This is consistent with work from our group and others providing evidence for sequestration of these proteins by repeat-RNA in *C9ORF72*-ALS cases (Cooper-Knock et al., 2014, 2015a). Moreover, we identified rare deleterious variants in these proteins in patients without *C9ORF72* expansions suggesting that dysfunction of G4C2 binding partners could be pathogenic in the absence of *C9ORF72* expansions. Other mechanisms of *C9ORF72*-ALS pathogenesis have been highlighted in the literature, but our findings support the relative importance of the repeat-RNA sequestration hypothesis. We have shown that, based on proposed RNA toxicity, we could select candidate genes and identify novel ALS genetic variants.

It is noteworthy that if all identified mutations are considered together then there is no correlation between variant-load and clinical phenotype. This probably reflects the diversity of mechanisms affected. To understand oligogenic inheritance, our data suggest that mutations will have to be understood as acting synergistically only within groups of functionally related genes/proteins.

Many of the variants identified potentially represent novel causative ALS genes, but we were not able to demonstrate segregation in families due to an absence of available samples. In certain cases the clustering of mutations with changes identified in Project MinE and the ALS Variant Server is highly suggestive of true pathogenicity. Most compelling are examples where we have identified more than one patient with a candidate mutation. Mutations that we believe are most likely to represent novel ALS variants and genes will now be discussed.

### SLC1A3

*SLC1A3* encodes excitatory amino acid transporter 1 (EAAT1) which is a glial glutamate transporter and also a G4C2 binding partner. Mutations of *SLC1A3* are a cause of episodic ataxia type 6 (EA6). The proposed mechanism is excitotoxicity via loss of glutamate uptake—excitotoxicity has also been proposed as a pathophysiological mechanisms in ALS (Cooper-Knock et al., 2013). Of the mutations associated with EA6, a p.C186S mutation in transmembrane segment 4 is the closest to both of our identified variants: p.N216S and p.F170L (Table 4). Transmembrane segment 4 has been associated with inter-subunit contact to stabilize the trimeric structure of the transporter (Yernool et al., 2004). The p.N216S mutations occur in a eukaryotic specific insertion between transmembrane domains 4b and 4c. The p.F170L mutation occurs in transmembrane domain 4A. Interestingly, while complete loss of SLC1A3 function leads to a severe phenotype with progressive ataxia (Jen et al., 2005), mutation in transmembrane segment 4 has been associated with partial loss of function and variable penetrance (de Vries et al., 2009) which is consistent with a late onset disease such as ALS. It is noteworthy that Project MinE identified an additional ALS patient with a rare (ExAC frequency <1/10,000 control alleles) mutation within the 4A transmembrane region.

### EEF1G

*EEF1G* encodes a component of the elongation factor-1 (EF1) complex involved in the elongation phase of protein translation which is a G4C2 binding partner. The EEF1G subunit is not proposed to have a direct role in translation (Fan et al., 2010), but co-immunoprecipitates with tubulin (Janssen and Moller, 1988) and has been observed to bind mRNA directly (Al-Maghrebi et al., 2002). This is consistent with a role for EEF1G in anchoring and translation of mRNAs in cytoskeleton bound ribosomes (Corbi et al., 2010). Translation at sites distant from the nucleus is particularly relevant in neurons and in large motor neurons in particular. We have identified two patients with mutations in the C-terminal domain of EEF1G: p.D403E and p.R327C (Table 4). Project MinE identified an additional ALS patient with a T902C variant in exon 8, the same exon as the C979T change we have identified.

### XRCC6

XRCC6 is a component of the non-homologous end joining (NHEJ) complex involved in repair of double stranded DNA breaks and is a G4C2 binding partner. Two patients were identified with rare deleterious variants in XRCC6: p.M348T and p.G589R (Table 4). Both variants occur within DNA binding domains, therefore both variants could conceivably lead to loss of function which is consistent with our disease model. Deletion of *XRCC6* in mice leads to premature aging without an increased rate of neoplasm (Li et al., 2007). This is consistent with observations in ALS and indeed impairment of NHEJ has been previously implicated in ALS (Sama et al., 2014).

### PPARGC1B

PPARGC1B is a transcription factor with roles in energy metabolism and mitochondrial biogenesis and a RRM-containing protein with a PrLD. We identified three young sporadic patients with rare deleterious variants in *PPARGC1B:* pP385H, p.S589G, and p.E434K (Table 3). Two of the variants identified lie within exon 4 either within or close to a low complexity region containing glutamic acid repeats. The p.E434K variant is actually within the glutamic acid repeats region and the same genetic change is observed in an additional three familial ALS cases within the ALS Variant Server. It seems likely that the variants we have identified and those found in the ALS Variant Server affect the function of the PrLD within PPARGC1B, leading to an increased risk of pathological aggregation.

### C9ORF72

A rare predicted deleterious variant was identified in *C9ORF72* in a patient who also carries a G4C2-repeat expansion. From a single patient it is not possible to determine whether there was synergy between the variant and the expansion but it is noteworthy that the patient identified suffered rapidly progressive disease: death occurred in 12 months from first symptom onset. In our population this is within the 10% most rapidly progressive *C9ORF72*-ALS patients (Cooper-Knock et al., 2012). If this variant is pathogenic and synergistic with the G4C2-repeat expansion, then it provides some insight into the pathogenesis of *C9ORF72*-ALS. A variant in *C9ORF72* could not recapitulate the proposed gain-of-function toxicity attributed to the G4C2-repeat, but it could potentially cause loss-of-function highlighting the relative importance of proposed haploinsuffuciency due to G4C2-repeat expansion.

## Conclusion

For the first time we have provided evidence for an oligogenic model of ALS in which rare variants act synergistically within discrete pathways. We have highlighted RRM-containing proteins with PrLDs and illustrated how mutations in G4C2-binding partners might exacerbate sequestration of the same proteins by repeat-RNA transcribed from the *C9ORF72* expansion. Several of the mutations we identified are candidate novel ALS genes and we have highlighted the examples of *SLC1A3, EEF1G, XRCC6*, and *PPARGC1B*. Our findings have significant implications for the design of ALS disease models and therapeutics.

## Ethics statement

This study was carried out in accordance with the recommendations of South Sheffield Research Ethics Committee with written informed consent from all subjects. All subjects gave written informed consent in accordance with the Declaration of Helsinki. The protocol was approved by the South Sheffield Research Ethics Committee.

## Author contributions

JC-K, AH, GH, JK, and PS were responsible for the conception and design of the study. JC-K, PH, MW, TW, MK, CM, PI, and PS were responsible for data acquisition. JC-K, HR, and IN were responsible for analysis of data. JC-K, AH, GH, JK, and PS were responsible for interpretation of data. The Project MinE ALS Sequencing consortium was involved in data acquisition and analysis. All authors were responsible for revising the manuscript and approving the final version for publication. All authors are responsible for the accuracy and integrity of the work. All authors, including members of the Project MinE ALS Sequencing consortium, meet the four ICMJE authorship criteria.

### Conflict of interest statement

The authors declare that the research was conducted in the absence of any commercial or financial relationships that could be construed as a potential conflict of interest.
