# Supplementary figures and images for: Targeted Genetic Screen in Amyotrophic Lateral Sclerosis Reveals Novel Genetic Variants with Synergistic Effect on Clinical Phenotype
Source: Front Mol Neurosci. 2017 Nov 9;10:370. doi: 10.3389/fnmol.2017.00370 (PMC5684183; doi:10.3389/fnmol.2017.00370)

**Supplementary Figure 1. Variants validated by Sanger Sequencing.**

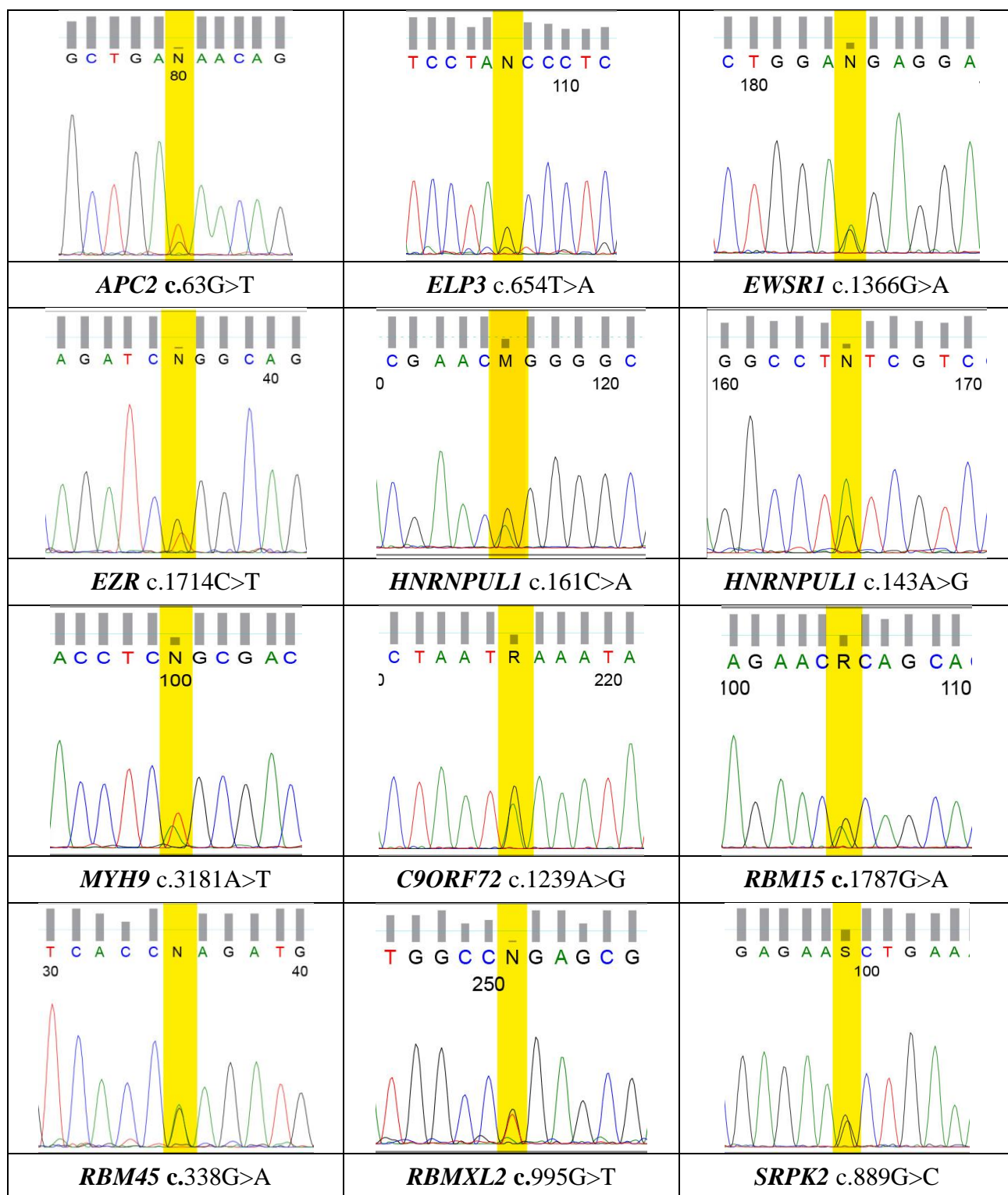

|                                                                                  |                                                                                   |  |
|----------------------------------------------------------------------------------|-----------------------------------------------------------------------------------|--|
| 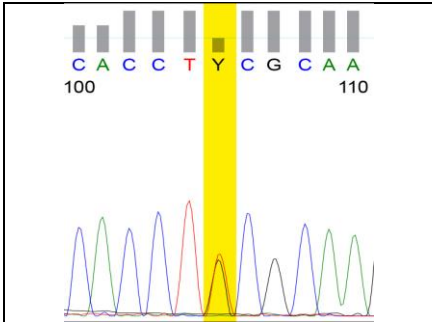 | 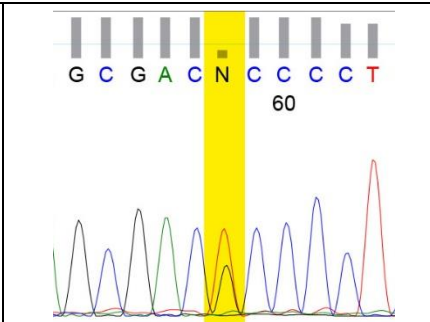 |  |
| <b><i>SYN3</i></b> c.1312C>T                                                     | <b><i>RBMXL3</i></b> c.362C>T                                                     |  |

Supplement: Supplementary file 3 [file Image1.PDF]
